# Ranking the university stars: are opinions sufficient?

**DOI:** 10.1007/s12471-014-0547-7

**Published:** 2014-03-26

**Authors:** E. E. van der Wall

**Affiliations:** Interuniversity Cardiology Institute of the Netherlands (ICIN) - Netherlands Heart Institute (NHI), P.O. Box 19258, 3501 DG Utrecht, the Netherlands

The Times Higher Education (THE) World University Rankings 2013–2014, endorsed by Thomson Reuters (USA), are the only global university performance tables to judge world-class universities across all of their core missions—teaching, research, knowledge transfer and international status. The top university rankings employ 13 carefully calibrated performance indicators to provide the most comprehensive and balanced comparisons available, which are trusted by students, academics, university leaders, industry and governments.

The top 10 THE World Reputation Rankings for 2014 are listed as follows: 1) Harvard University, 2) Massachusetts Institute of Technology (MIT), 3) Stanford University, 4) University of Cambridge (UK), 5) University of Oxford (UK), 6) University of California, Berkeley, 7) Princeton University, 8) Yale University, 9) California Institute of Technology (Caltech), and 10) University of California, Los Angeles (UCLA). This means that the top 10 consists of 8 universities of the USA and 2 from the UK. This pattern has been rather consistent over the past years.

However, a rejoicing fact is that for the first time in history, a Dutch University has entered the top-50: the Technical University of Delft (TU Delft) ranking 42 on the THE World Reputation Ranking list. Other Dutch universities on the THE World Reputation Ranking list (top 100) are the University of Amsterdam (no. 71), the University of Leiden (no. 84), and the University of Utrecht (no. 89). Altogether, a remarkable result for the TU Delft implying that the most prestigious university in the Netherlands is currently located in Delft. This a great achievement for TU Delft most likely due to its nature as a very specialised technical university, offering an excellent international profile in its area of expertise. It also may indicate that technical revolutions largely determine the progress on our globe.

According to the information provided on the THE website, http://www.timeshighereducation.co.uk/, the THE World Reputation Rankings 2014 employ the world’s largest invitation-only academic opinion survey to provide the definitive list of the top 100 most powerful global university brands. A spin-off of the annual THE World University Rankings, the reputation league table, is based on nothing more than subjective judgement, but it is the considered expert judgement of senior, published academics—the people best placed to know most about excellence in our universities.

As stated before, the THE World University Rankings offers detailed comparative performance information on the world’s top universities against the broadest range of a university’s activities, such as teaching, research, knowledge transfer and international status. To that purpose, each year over 10,000 of academics are invited to provide their expert opinion. These individuals are requested to list a top 15 of universities in their area of expertise. Important data points include acceptance rates, available financial aid, tuition fees, cost of living, the make-up of the student and faculty body, and dropout rates.

Consequently, the THE World University Rankings is only based on opinions of students and academics, no less no more. As a result, the outcome just defines the international reputation and with that the status of a particular university. Reputation and status are of course important in recruiting promising students and eminent academics, and obtaining funds and grants. This policy, however, harbours the risk of becoming a self-fulfilling prophecy due to positive feedback between belief and behaviour; once a university has been labelled as a centre of excellence, it will predominantly attract and select excellent individuals making the university more excellent: an upward spiral. This is the very reason that there are only marginal shifts in the top 50 over the years, making the achievement of the TU Delft even more special. Is an expert opinion sufficient for grading universities and ranking the stars? Generally, a scientific opinion or scientific consensus refers to the collection of the opinions of many different scientific organisations and entities and individual scientists in the relevant field. In that sense, opinions may herald important information, which may exceed that of factual and passionless knowledge. Is this the reason that we prefer to speak of opinion leaders instead of knowledge leaders?

